# Human Footprint Variation while Performing Load Bearing Tasks

**DOI:** 10.1371/journal.pone.0118619

**Published:** 2015-03-04

**Authors:** Cara M. Wall-Scheffler, Janelle Wagnild, Emily Wagler

**Affiliations:** 1 Department of Biology, Seattle Pacific University, Seattle, Washington, United States of America; 2 Department of Anthropology, University of Washington, Seattle, Washington, United States of America; 3 Department of Anthropology, Durham University, Durham, United Kingdom; 4 Arizona School of Podiatric Medicine, Glendale, Arizona, United States of America; University of Utah, UNITED STATES

## Abstract

Human footprint fossils have provided essential evidence about the evolution of human bipedalism as well as the social dynamics of the footprint makers, including estimates of speed, sex and group composition. Generally such estimates are made by comparing footprint evidence with modern controls; however, previous studies have not accounted for the variation in footprint dimensions coming from load bearing activities. It is likely that a portion of the hominins who created these fossil footprints were carrying a significant load, such as offspring or foraging loads, which caused variation in the footprint which could extend to variation in any estimations concerning the footprint’s maker. To identify significant variation in footprints due to load-bearing tasks, we had participants (N = 30, 15 males and 15 females) walk at a series of speeds carrying a 20kg pack on their back, side and front. Paint was applied to the bare feet of each participant to create footprints that were compared in terms of foot length, foot width and foot area. Female foot length and width increased during multiple loaded conditions. An appreciation of footprint variability associated with carrying loads adds an additional layer to our understanding of the behavior and morphology of extinct hominin populations.

## Introduction

In the process of reconstructing the hominin lineage, every available clue has the potential to provide key insights into the timeline and pathway by which the hominin lineage has evolved. Skeletal elements have been the basis for the identification of newly discovered hominin species [e.g. *Ardipithecus ramidus* [1], *Australopithecus sediba* [2]] and provide insights into what specific and unique adaptations each species may have had, the intrapopulation diversity [3], and when particular human-like traits (e.g. increased encephalization and bipedalism) first appear in the fossil record. For all hominins, skeletal remains provide valuable insight into morphology, behavior and niche. For example, the stature of extinct hominins has been estimated based upon the reconstruction of the skeleton [4, 5], femur/stature ratio [6], and regression formulae [7, 8] with each method independently producing comparable results. Hominin body mass can be estimated based upon femoral head breadth [5, 9, 10], hindlimb joint size [11] or by stature and pelvic breadth [12], though for extinct hominins whose stature is an estimate, this is less reliable [13]. Furthermore, skeletal data allows the degree of a species’ sexual dimorphism to be determined [14, 15].

Fossilized skeletal elements also provide bases for interpretation of locomotor style and effectiveness, giving further clues as to niche and behavior. The functional morphology and locomotion of extant populations and individuals has been studied in depth, and from these studies the particular anatomical traits most essential for humans’ terrestrial bipedality (e.g. lumbar lordotic curve, large femoral head, anteriorly curved iliac blades) have been identified [16, 17]. By looking for these traits in extinct hominin skeletons, extinct hominins’ capability for human-like bipedality can be estimated, though how species-specific traits not seen in humans (e.g. broad iliac flare in *A. afarensis*) affect locomotor style is not agreed upon [18–20].

A possible means of bridging gaps between the morphology and locomotor effectiveness lies in evidence from fossilized footprint trails. Fossilized footprints from a broad range of time periods [Laetoli [21], Ileret [22], Hawaii [23]] have augmented skeletal data by providing additional evidence corroborating and/or reshaping the interpretation of the skeletal data of a particular species. Footprints have been used to supplement stature and body mass estimations based upon skeletal segments because forensic data show quite clearly that footprint dimensions correlates well with stature [24] and unloaded body mass [25]. Additionally, particular aspects of foot morphology can be directly observable within fossilized prints and compared with reconstructions based on bony elements alone (e.g. toe pad shape and separation, toe length, and hallux position [22, 23]). Finally, specific details of locomotor patterns are preserved in fossilized footprints. Footprints have been used to indicate the cadence at which the species walked [17], the stride lengths of the individuals [23, 26–30], the pattern of weight transfer while walking [31, 32] and whether individuals were walking with other individuals [21, 22]. Experimental data from extant populations with recorded stature, mass, cadence and speed can further confirm relationships between the footprint tracks and behavior of extinct hominins [31, 33–35]. Thus footprint data have the potential to add crucial dimensions to paleoanthropologists’ interpretation of functional morphology and enhances understanding about extinct species’ locomotor and social behavior.

The first direct evidence of habitual bipedality comes from the Laetoli footprints (3.6mya) [36], most likely made by *Australopithecus afarensis* individuals [37]. While it cannot be certain what particular subsistence strategy these extinct hominins used, it has been strongly suggested that they adopted a form of foraging subsistence [38]. It is thus not clear how far their daily range may have been (and there is some disagreement) [39–41], though it has consistently been suggested that early bipeds carried some amount of foraging or lithic load in addition to carrying young offspring [42–53]. Therefore, because load-carrying of infants is a primate norm and carrying some amount of subsistence or technological product was likely typical of bipedal foragers [54], it is possible that the interpretation of some of the morphological characteristics of footprint makers, particularly mass and stature, will be influenced by the presence of the additional load.

How these populations carried the burdens is another important question, as different burden locations change both the mechanics and energetics of walking differently [49, 55–59], and thus will have different stabilizing requirements which may influence footprint pattern [60]. Load carrying comes with some amount of postural adjustment, specific to the position of the load (e.g. a load on the left will come with a torso tilt to the right) [61]; such postural accommodations are accompanied by footfall adjustments including changes in stride length, contact time, step width, and speed or velocity [55, 59, 61–64]. These changes in kinematics have been shown to have significant effects on footprint patterns as well [33, 35, 36] suggesting that burden carrying, and carrying position in particular, may have an influence on the behavior of the foot during stance phase [65].

Ethnographic data on foragers suggests that women generally carry young babies on the front [66–68], and obviously the load of pregnancy (between 15–25% of body mass) is also located anteriorly. Older babies are often carried on the side while foraging [69], and some women have side satchels to load with tubers and other plant resources [70]. Additionally, mixing locations is typical and Ache women carry belongings and food on their back, and their children on their side [71]. Data on the !Kung also show diverse patterns of carrying, with a pouch on women’s backs for food, water and firewood, and various types of side satchels for tool kits, babies, and larger food items; older toddlers may also be placed in the pouch on the back [70]. Many women also carry plant resources on their back via a tump-line [51, 59, 72, 73] or other back basket [74].

!Kung and other San men primarily use side satchels, with differently sized satchels for their tool kits (smaller) and food (larger) [69, 70]. Men across cultures may also use their digging sticks to create large yokes carried between two men for the purpose of carrying meat or bags of tobacco [70, 75]. Men also carry meat slung over their torso in large strips [75] or sling the meat across their shoulders, with the mass of the meat located either anteriorly or diagonally-posterior to anterior [74]. Meat and tools may also be carried in their hands. Among the Pumé, men also use a carrying pole, but each man will carry his own, placed over a single shoulder with balance weight slung from each end [76]. Periodically men will also carry on their back using a tump-line/head-yoke [59, 73].

It is thus clear that there are sex differences in loading behavior [51, 55, 71, 77–81], as well as in loading placement. There is further evidence that females tend to carry burdens of a larger amount of mass, both relatively and absolutely, in the majority of hunter gatherer populations [51, 77, 80, 81, 92, 102]. The importance of understanding the role that such patterns of behavior have on footprint morphology seems integral then to the understanding and interpretation of fossil footprints and other aspects of morphology.

Additionally, studies on the interaction between speed and burden suggest that carrying burdens significantly influences walking speeds, generally by making them slower if that is an option [55, 71, 80, 82, 83]. However, additional research shows that when traveling long distances and/or traveling quickly, women are more likely to carry heavy, older offspring [84, 85]. This suggests that the interplay between burden and speed may be moderated by other aspects of daily life, including transport distance [51]. Since both burden and speed have clear interactions when it comes to locomotor energetics and biomechanics [55, 86] and since speed alone has been shown to have such a significant relationship with footprint dimensions [33, 36, 87], it seems that any investigation into the importance of burden carrying should also consider the speed at which the carrier is moving.

In order to investigate the interactions between speed and load on linear footprint patterns, here we test the effects of a 20-kilogram load on footprint shape when compared to prints made by the same individuals in an unloaded condition; both the unloaded and loaded conditions are carried out at three different speeds. Based on ethnographic literature, 20kg is well within the range of the mass of carried carcasses [88–90], nuts [80, 91, 92], plants [51, 69, 71, 73, 77], or young children [71, 84] carried by foraging populations. Twenty kilograms is also the amount attributed to raw material loads for early hominins at Olduvai [53]. Furthermore, when people are carrying their children (upwards of 4 and 5 years old in some foraging populations) [93], they are often carrying the additional loads of plants and nuts in particular, making 20kg a reasonable load assessment for both males and females.

It is clear based on studies of gait, ethnographic information, and the fossil record that we needed to collect a footprint series at each condition, and not a single footprint or pair of footprints. Since gait changes are numerous and potentially variable [33, 36, 55, 59–65], only in seeing an entire gait sequence can we assess the potential changes in footprints caused by these different loading conditions. This requirement precluded us from using a foot pressure plate that would only have captured a single foot fall in one gait sequence. While this is often accommodated in the literature by simply including many trials at the same condition, in order to compare with naturally occurring footprint tracks, we were interested in assessing the variation across an entire track of footprints made at one time. Additionally, when people perform novel tasks, or tasks in novel places, as might happen when carrying a load, it is possible for certain posture changes to occur [94, 95], so we wanted to ensure that our participants were in a place familiar to them, thus choosing a hallway in one of the major classroom buildings on campus (though after hours, so other people were not a distraction). Finally, we had to decide whether to use a forcepad runway (“carpet” walkway), or the paper-and-pen method of measuring footprints. Both methods have been used in a variety of fields [96, 97], and primarily for the purpose here, namely to measure foot width, foot length, stride length, and stride width across a gait series. For the measures we are taking here, it has been shown there is no difference between the paper-and-pen method and the carpet walkway [98]. That being said, some problems with the carpet walkway methods include having to step very hard in order to get the ‘correct’ responsiveness [98] (that is, a full footprint image) and the possibility of people changing their gait due to the electronic (vibrating) nature of the walkway [94]. To this end then, we decided on the paper-and-pen method of footprint analysis.

## Methods

Thirty individuals (15 males, 15 females; age range 20–27 years, mean = 21.6years) were recruited for participation in this study. This research was approved by the IRB of Seattle Pacific University. The IRB approved the entire protocol as described in the paper. Every participant signed written informed consent, approved by the IRB, which completely explained the approved protocol. Our sample is representative of the general population as males were heavier than females (respective means of 84.5kg versus 70.8kg) and taller than females (respective means of 182.8cm versus 167.7cm).

All participants were healthy and exhibited no unusual or pathological foot morphologies or gait patterns. The participants represent a habitually shod population. While shod and unshod people do show differences in footprint patterns, one of the features of habitually shod feet is decreased foot flexibility [99], suggesting the feet will have reduced displacement [100, 101] and that we are less likely to find variations due to load bearing conditions; however, even stiffer feet may exhibit displacement if the load is heavy enough [101]. Thus we do expect that the sample here should suit our purpose of looking at loads.

As ethnographic literature contains numerous evidences of load carrying behavior, we chose to look at the most consistently used loaded positions: unloaded, front-loaded, side-loaded, and back-loaded. Additionally, since previous research has shown that speed influences footprint patterns generally by increasing print length [33, 36, 87], at each loaded condition, we had participants walk at three self-selected walking speeds (slow, medium, fast). These 12 conditions were randomized for each participant using a random order generator (http://www.random.org).

### Loading conditions

A 20kg load was carried for each loaded trial. This load represented on average 23.7% of male body mass and 28.2% of female body mass. While is seems clear that the maximum load possible for a person to carry is influenced by his or her own mass and musculature, typically people carry absolute loads and are not necessarily able to temper the load based on their body mass. The results from the ethnographic literature on foragers consistently state absolute burdens of food, children and household goods carried great distances particularly by females [51, 77, 80, 81, 92, 102]. This literature further suggests that females carry higher absolute and thus relative loads than males. To this end we felt it would be more realistic to assess the footprint behavior across males and females while they carried an absolute load.

In the front-loaded trials, a backpack loaded with a 20kg weight was worn backwards so that the weight sat at the belly. In the back-loaded trials, the same 20kg backpack was worn traditionally with the weight sitting at the middle of the back. In the side-loaded trials, a messenger-style bag loaded with a 20kg weight was worn across the body on whichever side each participant preferred based on their handedness and rested on the side of the hip between the ilium and greater trochanter. Both the backpack and messenger bag were adjustable and were adjusted to fit each person’s stature to lie at the same belly/back/hip locations. In the unloaded trials, participants walked with no bag and no load.

### Speed conditions

The three walking speeds were self-selected by each individual. Immediately prior to the testing session, participants were given a series of speed directives. That is, they were told to determine what they thought was a slow walking speed (as slow as possible while still maintaining a walking gait), a medium walking speed (a speed they would probably choose when walking normally), and a fast walking speed (as fast as possible while still maintaining a walking gait). Participants were given as much time as necessary to get a feel for each of the speed directives so that they could consistently replicate them during testing. During each condition, the actual walking speed was determined using a stopwatch beginning at the first moment of the dynamic heel-down to the moment the person left the paper. This was then divided by the measurement of the distance that they traveled (see measurements below). The stopwatch was operated by the same person for all trials and participants to ensure consistency. As the runway was short (∼5m), the person running the stopwatch had a clear view of the entire runway and could accurately assess the start and the stop; the use of a stopwatch to accurately determine time in walking trials is common [98, 103].

### Data collection

Six sheets of white butcher paper (4.6m long by 0.7m wide) were laid atop a stone floor that was uniform in texture. Immediately prior to each walking condition, participants sat in a chair at the end of each “track” while non-toxic colored paint diluted with water in a 1:1 ratio was applied to the bottom of both feet with a foam paintbrush. The paint was mixed and applied by the same person for all trials and participants to ensure consistency.

**Fig 1:**
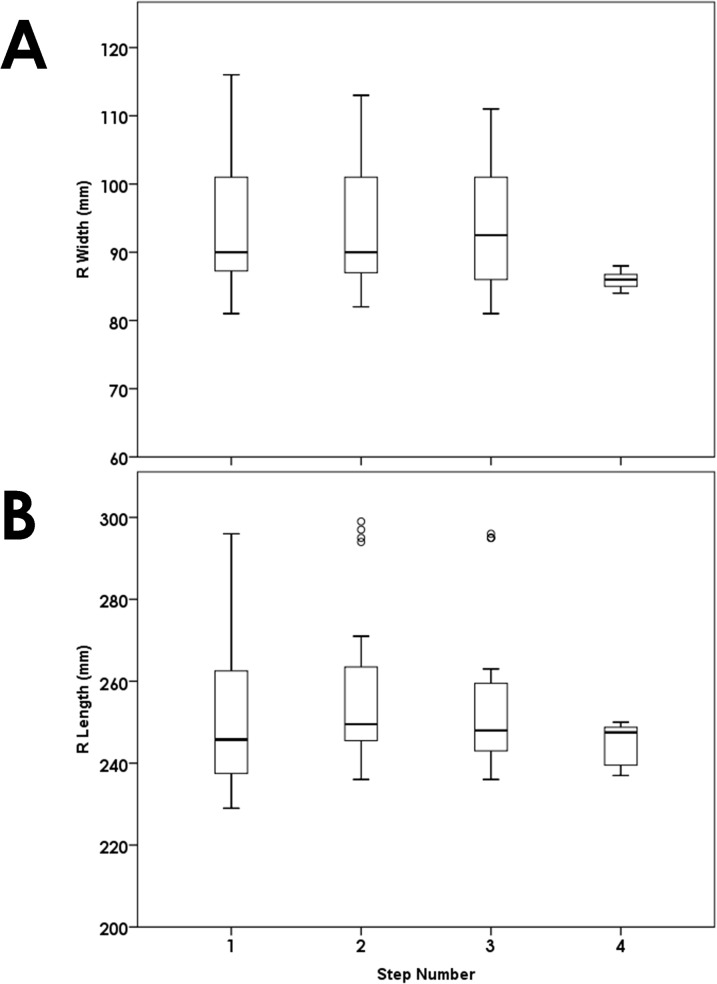
There was no influence of changes in paint amount or consistency across each trial: between step width and step number, p = 0.328; between step length and step number, p = 0.959.

Once the paint had been applied, participants stood with feet shoulder-width apart at the end of the track and were loaded if applicable. They were then asked to take a step forward with both feet and hold for about two seconds to create a set of static footprints, and then were told to walk through the end of the track onto towels laid beyond the ends of the track according to the speed cue they were given. Most people made three footprints for each foot, with some people making four (total footprints between 6–8). There was no significant relationship between the footprint measures at the first step and the subsequent steps, suggesting that any potential ‘loss of paint’ over the course of each trial had no influence on the footprint measures (Fig. 1). All of the twelve, randomized conditions were performed within a one-hour period.

This ‘paper and pen’ method for creating the footprints was based upon protocols commonly and reliably used [35, 96, 104–107]. The paper and pen method on floor surfaces has widely been tested in terms of its validity, reliability and consistency [98], mostly recently against other ‘carpet’ walkway methods [108]. It is generally considered the ‘gold standard’ in terms of the measures applied here [98], even though the analysis is not automated. Besides reliability, the pros of the paper and pen method are that people are more likely to use their habitual gait without modifying it due to the unfamiliar circumstances (e.g. novel environment, texture)[109, 110] or by synchronizing with the electronic and oscillating resonance of the force plate [94]. While of great import for any locomotion study, these benefits are limited by the fact that we could not have identified specific moments of footfall timing (e.g. contact time) during the gait cycle. Such gait related variables are more ably attained through the use of something like a carpet walkway or force platform method; since these variables were not of interest here, we were able to preference the attainment of as natural a stride as possible.

We furthermore performed a small-scale (N = 10) reliability assessment and compared our paper and pen technique between footprints made on the floor and footprints made on a 6.4mm deformable latex pad. There was no significant effect of surface-type on the measures discussed below (p = 0.885), and the patterns between unloaded and loaded footprints were the same.

### Measurements

**Fig 2:**
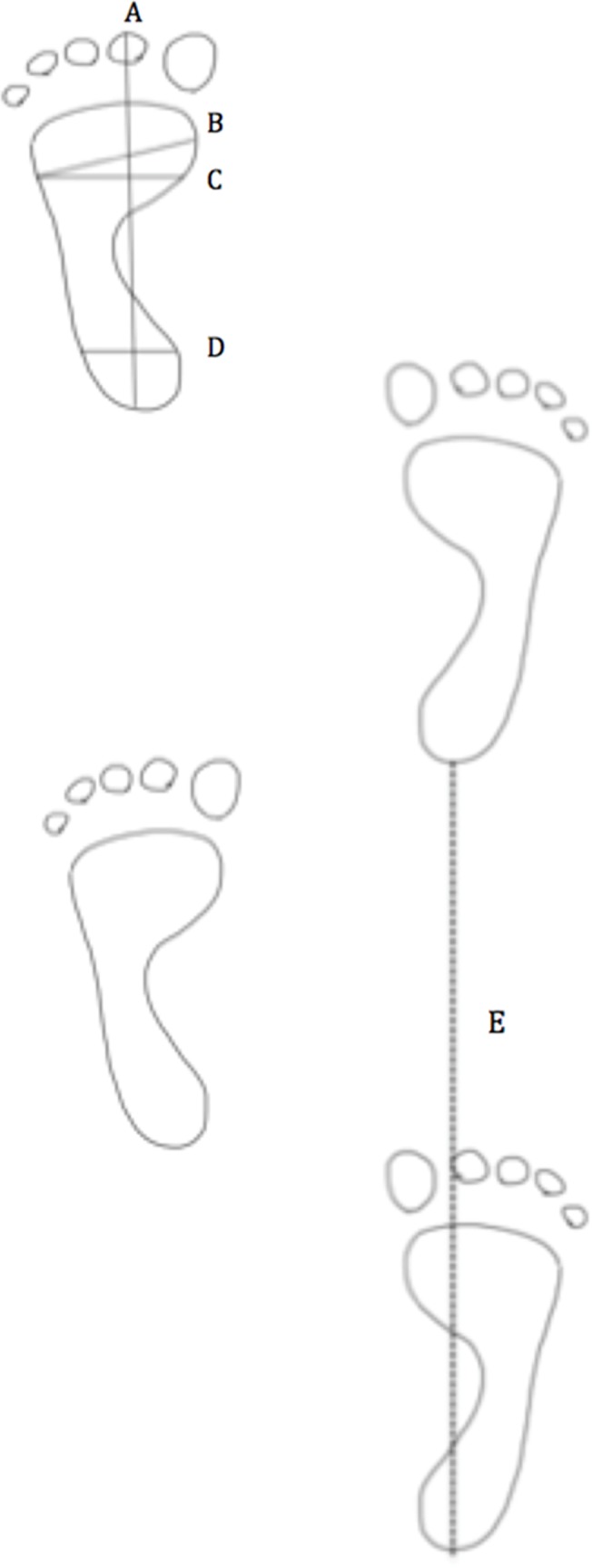
Maximum foot length (MFL) was measured as the distance along the Brannock axis: pternion to tip of second toe (A). Maximum foot width (MFW) was measured as the most lateral aspect of the footprint (fifth metatarsal) measured obliquely to the most medial aspect of print (B). For each series of footprints, stride length (E) was measured as the distance between the most posterior aspects of subsequent heels on each side.

Individual footprints were measured following Witana et al. [111], using the standard footprint measures of people measuring footprint remains [33, 35, 36]. The measurers were blind to all information (i.e. sex, load, speed) regarding the feet they were measuring. Maximum foot length (MFL) was measured as the distance along the Brannock axis: pternion to tip of second toe (Fig. 2). Maximum foot width (MFW) was measured as the most lateral aspect of the footprint (fifth metatarsal) measured obliquely to the most medial aspect of print; (Fig. 2). Foot area (FA) was calculated by multiplying foot length and foot width [33]. All feet were measured on both the right and the left sides. All data are available in S2 Dataset.

### Statistical Tests

All analyses were done for both the left and the right sides to verify stability of results. P-values and figures are given for the right side.

Based on previous research on sex differences in locomotor mechanics [112, 113], we expected sex differences in footprint patterns and thus collected on a large enough number of each sex that we had the power necessary to run the analyses for each sex separately if the differences proved significant. Since many studies pool the sexes even in unloaded conditions (expecting that controlling for mass will account for any differences), we investigated how much of the variation between the sexes in the unloaded condition alone was due to size (i.e. mass and stature), and how much might be due to sex-specific differences in foot morphology (see S1 Dataset). We thus ran a GLM multivariate analysis with MFL, MFW and FA as dependent variables, sex as a fixed factor, and mass and stature as covariates. Following this, we further investigated whether the sex-specific differences would also influence footprint behavior whilst loaded; to this end we repeated the above analysis, but included loaded versus unloaded as an additional factor.

As the positional difference between the three loaded conditions represents a factor of unknown influence on footprint patterns compared to unloaded walking, we ran a separate GLM multivariate analysis for each of the three loaded conditions, using loaded versus unloaded as a fixed factor in each case. All three footprint variables—MFL, MFW and FA—were included as dependent variables. Speed directive was furthermore included as a fixed factor; the results for speed directives are given separately, rather than as part of the loaded condition models, as the results were comparable across all of the loaded models.

## Results

### Sex

**Fig 3:**
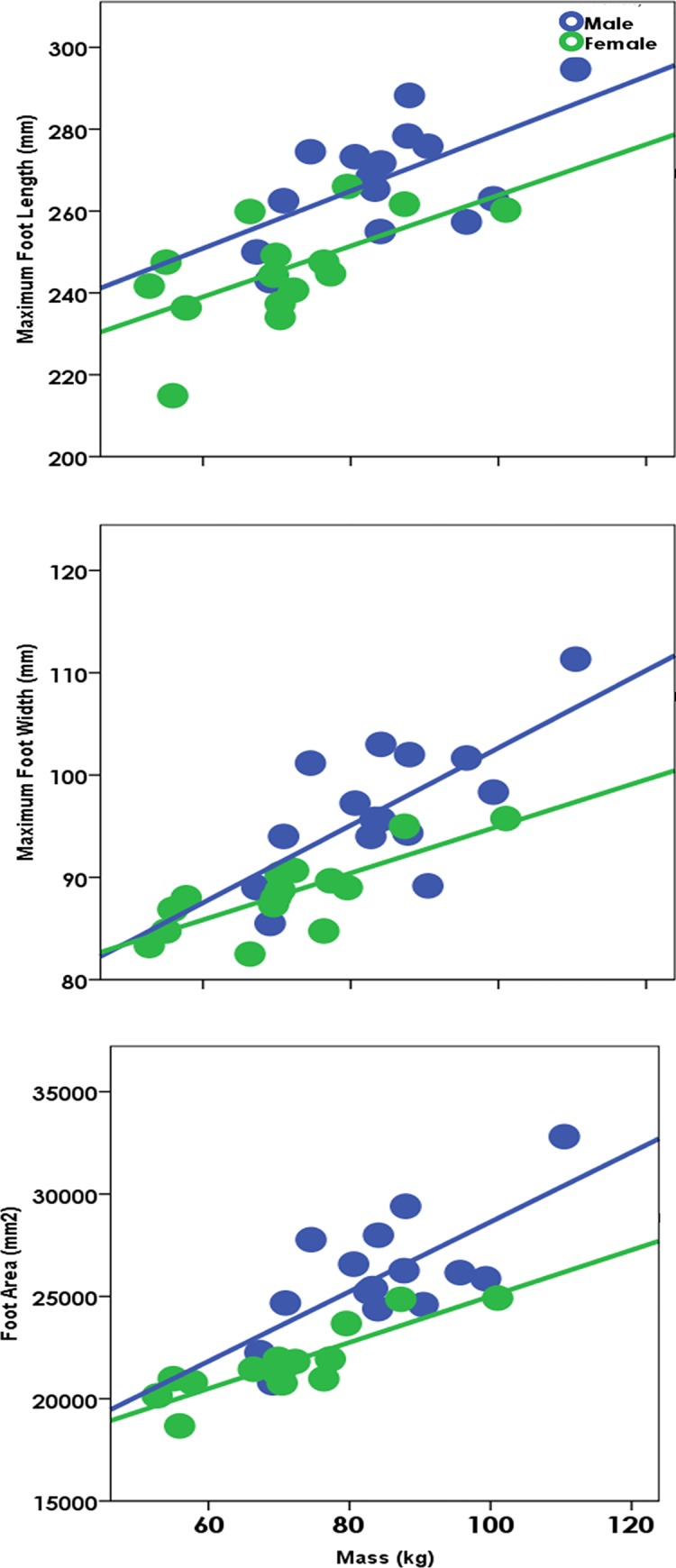
The significantly different mass-specific footprint patterns of males and females. These figures are for unloaded walking at the medium (comfortable) speed, but the graphs and patterns are identical for all speed conditions. The p-values for the residuals of the sex differences of these relationships are p<0.04 for maximum foot length; p<0.04 for maximum foot width; p<0.02 for foot area.

The GLM multivariate analysis of sex differences during unloaded walking on the three foot variables (MFL, MFW, FA) showed a significant sex effect (p<0.001) after mass and stature (p<0.001 for both) were included in the model. The effect of sex was strongest on MFL (p = 0.001) (Fig. 3). The GLM multivariate analysis of sex differences during loaded walking on the three footprint variables similarly showed a significant sex effect (p<0.001). Based on these two results, we were obligated to investigate the influence of load on footprints for each sex separately.

### Front Load

**Fig 4:**
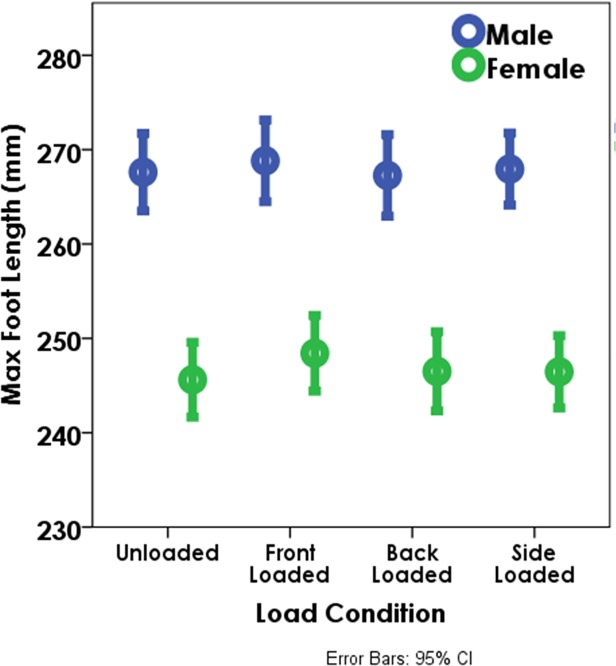
The increase in maximum foot length (MFL) at each of the loaded conditions by sex (all speeds combined). The circles denote the means and the error bars represent the 95% confidence intervals. MFL was consistent across all loading conditions for males. For females, the difference between the unloaded and front loaded condition is significant (p<0.01).

**Fig 5:**
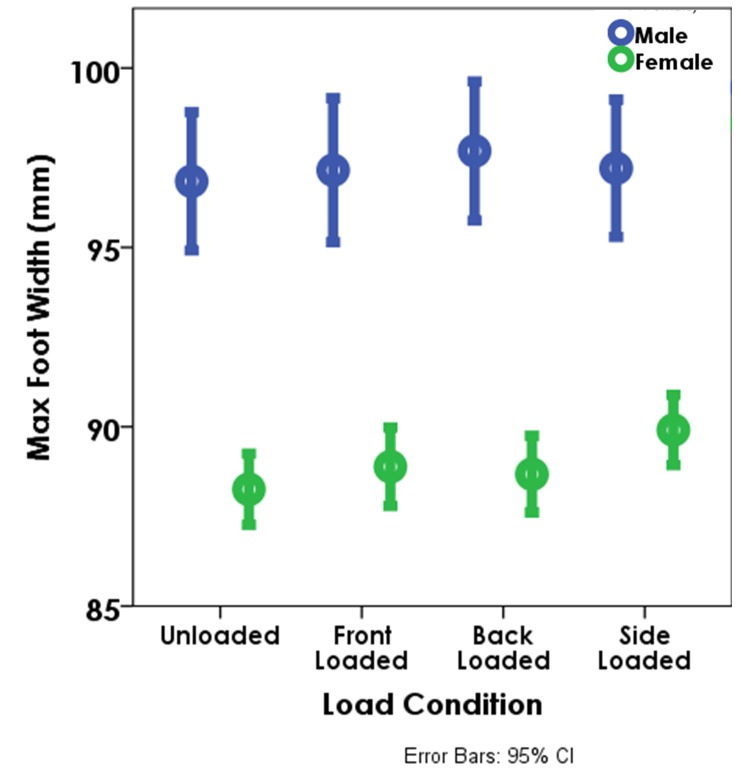
The increase in maximum foot width (MFW) at each of the loaded conditions by sex (all speeds combined). The circles denote the means and the error bars represent the 95% confidence intervals. The differences between the unloaded, and front and back loaded conditions are significant for both males and females at the slower speeds (p<0.05). MFW is also significantly larger for females between the unloaded and side loaded condition regardless of speed (p<0.05).

Females show an increase in footprint dimensions with a front load (p = 0.093 for load’s effect on the model) (Figs. 4 and 5); of the individual variables, FA reached significance (p = 0.042). Males show no significant changes in any footprint variables with front load.

### Back Load

Neither male nor female footprint variables show any significant changes with back load (Figs. 4 and 5).

### Side Load

Females show a significant increase in MFW with side loads (p<0.001) (Fig. 5) and as a consequence an increase in foot area (p = 0.018).

### Speed Directive

MFL increased with speed directive (e.g. “slow” or “fast”), though more clearly for males (p = 0.002) than females (p = 0.028). Speed directive had no significant influence on MFW or FA (See Summary in Table 1).

**Table 1:** A summary of significant changes (p<0.05) to footprint size and stride length during loaded walking.

	Speed	Load
**Maximum Foot Length (MFL)**	Increase	Increase for Females
**Maximum Foot Width (MFW)**	No change	Increase for Males and Females
**Foot Area (FA)**	Minimal change	Increase for Females
**Stride Length**	Increase	Decrease for Males and Females

## Discussion

**Fig 6:**
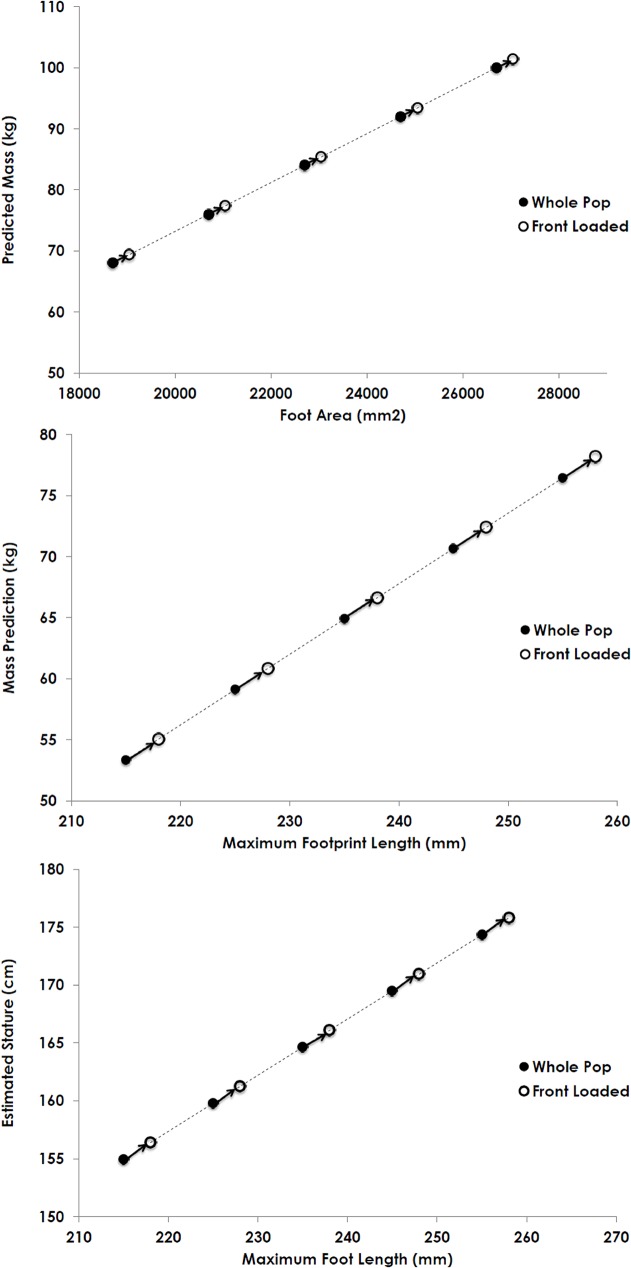
Showing the regression equations for maximum foot length and foot area predicting either mass or stature. Following the arrows from each point illustrates the overestimation of mass from footprints of loaded individuals, as determined by the mean percent increase.

These results indicate that both sex and load can significantly and substantially influence features of footprint shape and can possibly confound evidence as to the size of the footprint maker. Because of the different relationship between mass and footprint pattern for the sexes (Fig. 3), single population regression lines (regressions that include both sexes) will not correctly estimate the mass or stature of females in particular. Additionally, given the increase in footprint size with loads, the footprints of loaded individuals will lead to an overestimation of the mass and stature of the footprint makers (Fig. 6).

The impact load has on footprint shape and walking speed is heavily influenced by the loaded position. Though variable primarily based on speed, frontal loads generally increase the length of footprints in females and as a consequence the entire foot area—particularly relevant since non-contraception-using females will spend a reasonable proportion of their reproductive lifespan frontally loaded [114]. Interestingly, pregnant women generally experience an increase in foot width rather than foot length, at least in terms of absolute foot size (Wall-Scheffler, unpublished data). This finding here may have something specific to do with the stability during gait; by the postural and locomotor adjustments made to accommodate frontal load placement; as well as by therelaxation of tendons and ligaments during pregnancy [115]. When a load is placed on the front, the tendency is to lean backward in order to keep the center of mass in the midplane of the body [61]. This postural adjustment would reasonably cause a change in force distribution of the foot, resulting in the longer print. Additionally, frontal loads can cause shorter stride lengths and slower walking speeds [55]. Both of these factors resulted in increased contact time between the foot and the ground (usually because the body is working harder to generate the increased force necessary to perform a more challenging task) [86, 116–118], and likely result from changes made to minimize instability [116], another possible contributing factor to the enlarged prints. If the foot is provide stabilizing support, its ability to act as a propulsive lever may require additional pronation changing the width of the footprint.

Side loads have the largest effects on footprint shape, and are also one of the most common loading types among foraging populations [69–71, 76], used for both offspring and foraging loads. Side loads have been shown to decrease the stability of walking due to the asymmetrical nature of the load [49, 64] which is the likely cause of the footprint changes seen here. As with front loads, stride length can decrease with side loads and stride frequency will increase; additionally, with side loads, the angle of the trunk away from the midline is higher than while unloaded, and the trunk’s range of motion is greater—further supporting an idea of instability [64]; such instability may be potentially moderated by the footfall pattern [60] and lead to the wider footprints seen here, particularly among women. Back loads had minimal influence on footprint shape and may represent a loaded condition for which humans are well-suited [50, 57, 119], though some kinematic changes with particularly large back loads have been noted [116].

Loads influence the footprints of both males and females but seem to have a particularly substantial effect on female footprint morphology. Since both sexes carried the same mass (20kg) but had significantly different body masses, it may have been possible that the task difficultly drove the sex differences in foot morphology. However, the difference between the percent body masses of the load was small (4.5%) and footprint variation between the sexes exists even in the unloaded position (Fig. 3). Another factor instead may be foot composition and shape relative to other body proportions. Females have significantly shorter feet relative to both stature and lower limb length than males [120] (Table A in S1 Dataset). Because of this, females are placing a greater load (body plus load mass) on a relatively smaller surface area while walking, which may lead to the increased displacement of female foot area [60]. Additionally, males have been shown to have stiffer heels [100] and significantly more relative lean mass (relative to total foot mass; Table A in S1 Dataset) than females who have significantly more relative fat mass (relative to total foot mass; Table B in S1 Dataset) in their feet. Thus the greater percentage of fat tissue in female feet and the smaller feet relative to stature would render female feet more likely to undergo deformation during walking.

The greater proportion of fat in female feet may be another contributing factor to the growing body of evidence that females are efficient and economical load carriers [51, 55, 76, 78, 121]. According to Zelik and Kuo [122], soft tissue deformation—particularly soft tissue like fat and ligamentous connective tissue—can actually do work (elastic tissue rebound) in addition to saving muscles from having to actively dissipate energy. Zelik and Kuo [122] suggest then that fatty tissues are crucial both in cushioning impact and protecting damage and stress, as well as in performing work. Since females have more fatty tissue in their feet (and throughout their lower limb) and since this fatty tissue serves to conserve energy upon foot contact with the ground, female feet have an energetically efficient construction that may contribute to reduce the amount of energy spent when walking and/or carrying loads (along with other morphological adaptations; see [55]).

The following features thus indicate the importance of load carrying in hominin evolution, and the likelihood that the makers of fossilized footprint tracks were carrying loads: morphological adaptations that allow females to effectively carry loads [49, 55], the evidence that females throughout the hominin lineage have been carrying (at least) their babies [48, 50, 84, 123], and the fact that female feet have an increased proportion of fatty tissue [100] (Table B in S1 Dataset).

It should be noted that though significant, many of the changes in footprint size and shape fall within the percentage error of 1.7–14.5% noted by Dingwall et al. [33] that can occur based on substrate alone. Since it is clear that on a stable substrate footprint dimensions increase with loads, it possible that on an unstable or variable substrate footprint dimensions increase in a consistent and/or interactive manner, and perhaps to an even greater degree than shown here. People running on sand show evidences of instability similar to those reviewed here for load carrying: including increased stance times and shorter stride lengths [124]. This suggests that kinematic and kinetic changes that elongate the footprint may be of even greater magnitude on variable substrate than the 1.7–14.5% suggested for unloaded walkers [33]. It will be helpful in the future to measure people carrying loads on varying substrates in order to better assess what differences are ‘meaningful’ to our reconstructions of the morphology and behavior of past populations. Ignoring the potential of substrate and load interactions to change the pattern of footprint dimensions leads to the possibility of erroneous conclusions about morphology and behavior.

Finally, we have suggested based on research comparing habitually shod and unshod walkers [99] that the differences seen here may be less than what would be seen in unshod walkers due to the increased foot compliance among habitually unshod walkers even though our load was heavy enough to have provided the stimulus for displacement [101]. Further work comparing the sexes in shod and unshod populations will hopefully illuminate whether these are in fact minimum changes.

## Supporting Information

S1 Dataset Contains the data, with methods and results, from DEXAs on 6 men and 20 women for the purpose of elucidating the sexual dimorphism of foot composition. This file also contains Tables A and B. Table A, Mean Anthropometrics. Table B, Tissue Composition of Right Foot.
(DOCX)

S2 Dataset Study Dataset contains the data used in the main analysis. (XLS)
