# Supplementary material for: Human Footprint Variation while Performing Load Bearing Tasks
Source: PLoS One. 2015 Mar 4;10(3):e0118619. doi: 10.1371/journal.pone.0118619 (PMC4349815; doi:10.1371/journal.pone.0118619)
Supplement: S1 Dataset — This file also contains Tables A and B. Table A, Mean Anthropometrics. Table B, Tissue Composition of Right Foot. (DOCX) [file pone.0118619.s001.docx]

S1 Dataset

Methods

6 men (mean age 32.3, standard deviation 9.8) and 20 women (mean age 22.5, standard deviation 2.1) participated in a body composition study using DEXA. They all signed written informed consent forms for the study, as approved by St Catherine University’s IRB Committee. All DEXAs were performed at the University of Minnesota’s Clinical and Translational Science Institute. Lean mass, fat mass and total mass of the right foot were measured during the DEXA scan. Additionally, stature, mass, lower limb length and foot length were collected. Lower limb length (LLL) (greater trochanter to lateral malleolus) and foot length (FL) were collected using an anthropometer.

Results

Men were significantly bigger than women in terms of their mass (p<0.001), stature (p=0.003), absolute foot length (p<0.001) and foot length relative to both stature (p=0.001) and lower limb length (p=0.008) (Table A). Men also had significantly heavier feet (p<0.001) and significantly more absolute (p<0.001) and relative lean mass (p=0.023) in their feet (Table B). Similarly to other body segments, females had significantly higher percent fat mass in their feet (p=0.019) (Table B).

Table A: Mean Anthropometrics

|  | **Sex** | **N** | **Mean** | **Std. Deviation** |
| --- | --- | --- | --- | --- |
| **Body Mass (kg)** | Male | 6 | 88.6 | 6.8 |
|  | Female | 20 | 62.0 | 6.3 |
| **Stature (cm)** | Male | 6 | 177.8 | 7.4 |
|  | Female | 20 | 163.8 | 6.5 |
| **Foot Length (cm)** | Male | 6 | 26.6 | 0.9 |
|  | Female | 20 | 23.2 | 1.0 |
| **FL:Stat** | Male | 6 | 0.150 | 0.00 |
|  | Female | 20 | 0.142 | 0.01 |
| **FL:LLL** | Male | 6 | 0.319 | 0.01 |
|  | Female | 20 | 0.297 | 0.01 |

Table B: Tissue Composition of Right Foot

|  | **Sex** | **N** | **Mean** | **Std. Deviation** |
| --- | --- | --- | --- | --- |
| Total Lean mass (g) | Male | 6 | 629.0 | 52.2 |
|  | Female | 20 | 414.7 | 56.5 |
| Total Fat mass (g) | Male | 6 | 290.7 | 39.3 |
|  | Female | 20 | 245.3 | 49.9 |
| Total Foot Mass (g) | Male | 6 | 1010.3 | 59.1 |
|  | Female | 20 | 725.3 | 70.2 |
| % Lean Mass | Male | 6 | 0.62 | 0.04 |
|  | Female | 20 | 0.57 | 0.06 |
| %Fat Mass | Male | 6 | 0.29 | 0.03 |
|  | Female | 20 | 0.34 | 0.06 |
